# Evaluation of Methods to Quantify Sialic Acid on Glycomacropeptide

**DOI:** 10.3390/foods14223939

**Published:** 2025-11-18

**Authors:** Madison L. Dirks, Joseph Hale, Eric Theiste, Owen M. McDougal

**Affiliations:** 1 Biomolecular Sciences Graduate Programs, College of Arts and Sciences, Boise State University, Boise, ID 83725, USA; 2 Protein Research Center, Agropur, Le Sueur, MN 56058, USA; 3 Department of Chemistry and Biochemistry, Food and Dairy Innovation Center, Boise State University, Boise, ID 83725, USA

**Keywords:** *N*-acetylneuraminic acid, glycomacropeptide, chromatographic, mass spectrometry, acid hydrolysis, enzymatic, colorimetric, fluorometric

## Abstract

Glycomacropeptide (GMP) is isolated from whey and used as an ingredient in phenylketonuria-safe foods because it does not contain phenylalanine. GMP is highly glycosylated and has several sites where *N*-acetylneuraminic acid (NANA) is bound. In the dairy industry, quantification of NANA from dairy proteins is accomplished by colorimetric, fluorometric, enzymatic, and chromatographic procedures; there is no uniformly accepted industry-wide standard method. In this investigation, NANA quantification methods were evaluated using GMP, and a comparison was made based on the length of time to complete the assay, protein-specificity, linearity, precision, and accuracy. From the methods evaluated, the chromatography protocol was determined to have the greatest benefit for use as a dairy industry standard to measure NANA on GMP. The average mass percent of NANA in 10 statistically independent replicates from a commercial GMP product was measured to be 6.18% ± 0.12%, with a relative standard deviation of 1.94%, which was the lowest of all the methods tested. The accuracy of the chromatographic approach was validated using spike and recovery experiments that provided an average recovery of 90.25%.

## 1. Introduction

### 1.1. Glycomacropeptide (GMP)

Glycomacropeptide (GMP) is obtained during the cheesemaking process when rennet, containing the enzyme chymosin, cleaves the peptide bond on κ-casein between phenylalanine-105 and methionine-106, coagulating casein proteins and leaving whey proteins in solution [1,2]. The resulting 64 amino acid whey protein does not contain phenylalanine (Phe), which makes it a usable source of amino acids for phenylketonuria-safe foods [1,3]. Patients suffering from phenylketonuria (PKU) have a deficiency or mutation in their phenylalanine-4-hydroxlyase enzyme, which is responsible for the breakdown of Phe [4,5,6]. In addition to being a source of amino acids, GMP also promotes favorable bioactivity including bone remineralization, anti-inflammation, metabolism modulation, and antibacterial, antiviral, and prebiotic effects [1,7]. The mechanism of bioactivity is not known, but current theories attribute the activity of GMP to the peptide, the attached sugars, or a combination of the two [1,8,9]. The antibacterial and antiviral effects from GMP ingestion, as well as the inhibition of cholera toxin, are due to GMP binding to the bacteria, virus, or toxin, preventing them from binding to cells [1,10,11,12]. GMP binding is proposed to be directly related to the presence of the sugar *N*-acetylneuraminic acid (NANA) on the peptide [1,10,11,12]. NANA is the most abundant of a nine-carbon acidic family of sugars, referred to as sialic acids, that are found in humans and mammals. The extent of NANA glycosylation affects the functional properties of GMP, including foaming, emulsification, aggregation, gel formation, and solubility, which are important factors when formulating new food and beverage products [2,13,14].

### 1.2. NANA on GMP

**Figure 1:**
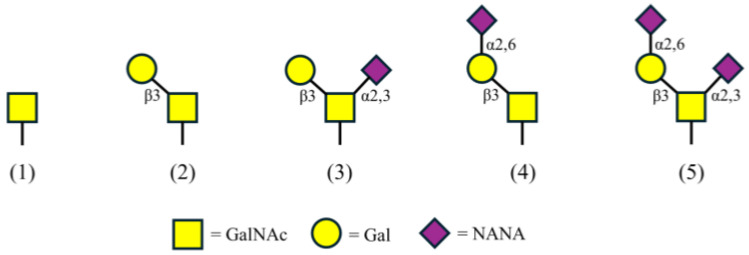
GMP’s five glycan structures composed of *N*-acetylgalactosamine (GalNAc), galactose (Gal), and *N*-acetylneuraminic acid (NANA).

GMP can be highly glycosylated with O-linked sugars that have five confirmed glycan structures (Figure 1) [15,16]. These carbohydrate groups are post-translationally added to serine (Ser) and threonine (Thr) residues including Thr-121, 131, 133, 136, 142, and 165, and Ser-141 and 142, among other potential sites [1,15,17,18,19]. The sugars are one to four units in length, composed solely of combinations of NANA, *N*-acetylgalactosamine (GalNAc), and galactose (Gal) [15,16,19]. With regard to GMP from bovine milk, the sites and level of glycosylation as well as the amino acid sequence vary with cow breed, lactation, and processing techniques [1,20,21,22,23]. Knowing the amount of NANA on GMP provides additional information to determine product value. NANA concentration can be used to properly adjust formulations for products requiring foams, gels, emulsification, or a soluble peptide source. This information can also be used to develop new products with health improvement claims, including cell protection via anti-adhesion of viruses, bacteria, or toxins. Measuring NANA on GMP can provide further opportunities and increased value for this glycosylated peptide. Establishing a standard method for NANA quantification is intended to lead to consistency in NANA measurement across the dairy industry.

### 1.3. Historical Methods for NANA Quantification

In order to measure O-linked glycosylation, the accuracy of the method is dependent on customization for the peptide/protein and sugar units targeted for quantification [23]. Sialic acids are found on the surfaces of both eukaryotic and prokaryotic cells, where they are used by viruses for binding, and they also occur in fungi [24]. The release of NANA from glycoproteins is influenced by the interaction of the physicochemical environment surrounding the sugar-connecting bond [23,25]. Many different methods have been attempted for quantifying NANA, but to date, there is no industry-standard method to accurately measure the amount of NANA on GMP. Methods to hydrolyze NANA from proteins utilize either the enzyme activity, neuraminidase or the chemical effect of acids, including hydrochloric, formic, sulfuric, acetic, or propionic acid [23,25,26,27,28,29,30]. Incomplete hydrolysis, detection interference from non-NANA sugars, and degradation of NANA due to its relative acid lability are common issues that limit the usefulness of NANA quantification protocols [23,29,31]. Enzymatic assays generally exhibit increased specificity, but occasionally their function can be disrupted by NANA’s bonding orientation and variation in O-acetylation and N-glycolyl groups [26]. Often samples require multiple enzymes to achieve complete NANA hydrolysis and transform NANA into detectable products [26]. Enzyme activity can vary from lot to lot, which can change the amount of NANA released from a sample, leading to poor accuracy for quantitation. Even when successfully used, enzymatic assays are expensive and time consuming [26]. When these methods were surveyed at Agropur, enzymatic assays yielded 0.25–4.11% (*w*/*w*) NANA on a single lot of GMP and acid hydrolysis assays reported 7.68–11.98% (*w*/*w*) NANA on a different lot of GMP. The lack of tight tolerance for results and poor accuracy to quantify NANA from GMP inspired the current work to develop a reliable method to measure NANA on GMP.

Once NANA has been hydrolyzed, colorimetric, UV, fluorometric, or chromatographic analysis can be employed for quantitation [26]. Colorimetric methods typically transform hydrolyzed NANA into an intermediate, which then reacts to form a chromophore to be measured spectrophotometrically [26]. These methods are subject to interference from hexoses, pentoses, 2-deoxyribose, and others, which limit their specificity for NANA [26]. Fluorometric methods often have similar, but longer, procedures to colorimetric assays, where NANA is hydrolyzed from a sample and then oxidized before reacting to form a fluorophore; in some cases the method will call for the addition of a fluorescent tag (such as 3,5-diaminobenzoic acid) instead of the fluorophore-forming reaction [26]. Some of the fluorometric assays produce the same toxic waste as the colorimetric methods and also show interference from hexoses, 2-deoxy sugars, aldehydes, and other compounds, but other fluorometric assays have far greater specificity to NANA [26,32]. Gas chromatography (GC) and high-performance liquid chromatography (HPLC) were studied using UV detection, and in some instances GC was paired with mass spectrometry detection (GC-MS) [26]. The GC-MS methods provided greater sensitivity, but also required derivatization and time-consuming purification processes, which lead to the loss of NANA [26,33]. Though many methods have been attempted for NANA quantification, there is still not an industry standard for GMP. The variety of methods employed give a range of results for NANA content on GMP, and some produce toxic waste, lead to degradation or incomplete release of the product, or have poor reproducibility [1,23,34]. The focus of the current investigation was to compare the four main types of experimental methods: colorimetric, fluorometric, enzymatic, and chromatographic, to determine which method is the most reliable, reproducible, and accurate to quantify NANA on GMP.

### 1.4. Current Methods for NANA Quantification

In 2017, an updated version of the Warren method was developed and packaged as a commercially available kit (Sialic Acid Assay Kit, MAK314, Sigma-Aldrich, Saint Louis, MO, USA) [34,35]. The Sialic Acid Assay Kit can be used for colorimetric or fluorometric detection of NANA, free or bound, within a sample of serum, plasma, saliva, milk, etc. [35]. For the hydrolysis of bound NANA, the sample is mixed with sulfuric acid and heated at 80 °C for 1 h. The freed NANA is oxidized to form formylpyruvic acid, which reacts with thiobarbituric acid, resulting in a chromophore that reflects red light and absorbs blue-green wavelengths at 549 nm using a microplate reader [35]. Fluorometric detection can measure NANA with ten times greater sensitivity at an excitation wavelength of 555 nm and an emission wavelength of 585 nm. The fluorometric assay uses the same workup as the colorimetric method, but the NANA standards used in the calibration curve are diluted 10-fold.

The enzyme α(2→3,6,8,9) neuraminidase from *Arthrobacter ureafaciens* cleaves glycosidic bonds attached to NANA [36,37]. The enzyme is available, along with several other reagents, in a commercially available kit (Sialic Acid Quantitation Kit, SIALICQ, Sigma-Aldrich, Saint Louis, MO, USA) for the measurement of NANA bound to glycoproteins, polysialic acids, or free in solution [36,37]. After enzymatic cleavage, NANA is converted to *N*-acetylmannosamine and pyruvic acid via *N*-acetylneuraminic acid aldolase [36]. The resulting pyruvic acid is reduced to lactic acid with lactic dehydrogenase and *beta*-nicotinamide adnenine dinucleotide (β-NADH) [36]. An indirect method of quantitation is used by measuring the remaining β-NADH post reaction [36]. Detection and quantitation of NANA can be achieved between 1 and 200 nmoles, using a microplate reader in the UV range at 340 nm.

Agropur US (Protein Research Center, Le Sueur, MN, USA) has developed an assay specific to GMP that employs weak acid hydrolysis in combination with high-performance liquid chromatography mass spectrometry (HPLC-MS) as a chromatographic approach to NANA measurement. This chromatographic method originated from non-satisfactory results obtained by experimental methods including the Warren method [34], enzymatic oxidation, and liquid chromatography with and without derivatization. A central composite Design of Experiments (DOE) was performed to determine the best hydrolysis conditions based on time, temperature, and acid concentration to fully remove NANA from GMP. MS detector drift was accounted for using internal NANA standards (C-12 and C-13). The Agropur chromatography method was internally replicated followed by third-party lab validation.

The purpose of the current project was to compare commercially available methods for measurement of NANA on GMP based on the time requirement for the assay, specificity of the hydrolysis method, the acid or enzyme, abilty to cleave NANA from GMP, linearity of calibration curves, precision of quantification, and accuracy of the assay.

## 2. Materials and Methods

### 2.1. GMP

Agropur produces a very high quality BiPRO^®^ GMP 9000 product (Agropur, Appleton, WI, USA) that is used in medicinal foods for individuals with PKU and as a functional ingredient in foods and beverages. The GMP tested in these experiments was lot number JE 0020-20-440. The certificate of analysis stated that it was 96.1% protein, 97.0% of which was GMP.

### 2.2. Colorimetric Kit

A commercial kit (MAK314, Sigma-Aldrich, Saint Louis, MO, USA) was used to measure NANA content from GMP obtained from Agropur US, by colorimetry, following the manufacturer’s technical bulletin and with additional guidance provided by Sigma-Aldrich technical support [35,38]. The NANA standard (10 mM) provided in the kit was serially diluted to provide concentrations of 1000, 600, 300, and 0 µM. The standards (20 µL) were combined with a 10% trichloroacetic acid (TCA) solution (5 µL) in microcentrifuge tubes in duplicate. For the hydrolysis reaction, a 20 µL aliquot of 600 µg/mL GMP was diluted with 40 µL nanopure (18.2 Mohm resistivity) water and mixed with 40 µL of hydrolysis reagent. Each sample was prepared in duplicate. The tubes were placed in a heat block set to 80 °C for one hr. The samples were allowed to cool to room temperature for 5–10 min before they were centrifuged at 14,000 rpm for 3 min. The 10% TCA solution (25 µL) was added to each sample and vortexed for 30 s. The tubes were centrifuged again at 14,000 rpm for 10 min. A working reagent was prepared in bulk for all samples and standards by combining the hydrolysis reagent (15 µL/tube), nanopure water (50 µL/tube), and oxidation reagent (65 µL/tube). Each sample (25 µL in a new centrifuge tube) and standard was combined with working reagent (125 µL) and allowed to sit at room temperature for one hr. For the color reaction, dye reagent containing 2-thiobarbituric acid (50 µL) was mixed into each tube of sample and standard. All tubes were placed in the heat block that was set to 100 °C, and heated for 10 min. The tubes were removed and allowed to cool for 5–10 min before being combined with dimethyl sulfoxide (DMSO) (100 µL), followed by centrifugation at 14,000 rpm for 5 min. For detection, samples and standards in aliquots of 250 µL were transferred to a clear, Greiner 96-well plate (Sigma-Aldrich, Saint Louis, MO, USA) and analyzed by Agilent BioTek Epoch 2 microplate spectrophotometer (Agilent, Santa Clara, CA, USA). Optical density (OD) was recorded at 549 nm. The OD values were averaged for all duplicates of standards and samples. The OD for the blank (0 µM NANA standard) was subtracted from the OD values of all other standards and samples. A calibration curve was generated with these corrected OD values and the reported concentrations. The following equation (Equation (1)) was used to calculate the concentration (µM) of NANA in each sample:

(1) $$\left[Sialic\;acid\right]=\frac{R_{sample}-R_{blank}}{Slope(\mu M^{-1})}\times n(\mu M)$$

where R_sample_ and R_blank_ are the OD or fluorescence intensity values of the sample and the blank, respectively, Slope is the slope of the calibration curve, and n is the dilution factor (5).

The concentration values (µM) were converted to NANA content (µg/mL) using the molecular mass of NANA (309 g/mol) and then to the mass percent (%*w*/*w*) by dividing by the mass of each GMP sample.

### 2.3. Fluorometric Kit

The same commercial kit (MAK 314, Sigma-Aldrich, Saint Louis, MO, USA) was used to measure NANA concentration by fluorimetry. The fluorimetry procedure was consistent with the colorimetric assay with a few exceptions. The NANA standards were prepared at 10-fold dilute concentrations of 100, 60, 30, and 0 µM. For detection, the samples were added to a black, clear bottom, Nunc_96-well plate and analyzed on a BioTek Synergy H1 Microplate Reader (Agilent, Santa Clara, CA, USA). Fluorescence intensity was measured using an excitation wavelength of 555 nm and an emission wavelength of 585 nm. In addition to subtracting the fluorescence intensity of the blank from all samples and standards, the fluorescence intensity of the empty plate was also subtracted from all wells.

### 2.4. Enzymatic Kit

A neuraminidase assay was performed using a kit (SIALICQ, Sigma-Aldrich, Saint Louis, MO, USA) by following the included instructions, and with additional assistance from the manufacturer [36,37]. First, a time-course experiment was conducted. The buffers and reagents were prepared from the stock solutions provided in the kit. The tris(hydroxymethyl)aminomethane hydrochloride (Tris-HCl) reaction buffer was prepared by diluting the 1 M Tris-HCl to 25 mM with deionized (DI) water. The β-NADH solution was generated by adding Tris reaction buffer (640 µL) to one vial of β-NADH (5 mg). A reaction mix was prepared with Tris reaction buffer (6510 µL), β-NADH solution (140 µL), *N*-acetylneuraminic acid aldolase (7 µL), and L-lactic dehydrogenase (7 µL). The reaction mix (1000 µL) was added to six microcentrifuge tubes, and what remained was reserved for a blank. For the initial time (T_0_ min), the GMP sample (40 µL of 600 µg/mL GMP in nanopure water) and sialidase buffer (10 µL) were added to one of the microcentrifuge tubes containing the reaction mix and incubated for one hr. in a water bath at 37 °C. For continuous digestion, the GMP sample (200 µL of 600 µg/mL GMP in nanopure water), sialidase buffer (50 µL), and α-(2→3,6,8,9)-neuraminidase (5 µL) were combined in a microcentrifuge tube and incubated in a water bath at 37 °C. Every hr. (for a total of five hr.), the sample (51 µL) was removed from the microcentrifuge tube and added to one of the microcentrifuge tubes containing the reaction mix. The reaction mix tube was returned to the water bath for an additional hr. before detection. The T_0_ sample, the blank, and each digested sample (200 µL in triplicate) were added to a clear, Costar 48-well cell culture plate and analyzed by absorbance at 340 nm using an Agilent BioTek Epoch 2 microplate spectrophotometer (Agilent, Santa Clara, CA, USA).

The enzymatic reaction began with mixing the GMP sample (40 µL of 600 µg/mL GMP in nanopure water), sialidase buffer (10 µL), and α-(2→3,6,8,9)-neuraminidase (1 µL). This mixture was prepared in duplicate and incubated for 4 h. in a water bath at 37 °C. Meanwhile standards of 1000, 600, 300, and 0 µM NANA were generated from the provided NANA standard (0.01 M). The Tris reaction buffer, β-NADH solution, and reaction mix were prepared in the same way as the time-course experiment with the volumes corrected for the number of samples being analyzed. After 4 h. of incubation, each sample (12 µL) was combined with reaction mix (238 µL) in new microcentrifuge tubes. Three blanks (250 µL reaction mix) were also added to microcentrifuge tubes. All samples, standards, and blanks were incubated for one hr. in a water bath at 37 °C. For detection, the blanks and samples (200 µL) were added to a clear, Greiner 96-well plate (Sigma-Aldrich, Saint Louis, MO, USA), and absorbance was measured at 340 nm on an Agilent BioTek Epoch 2 microplate spectrophotometer (Agilent, Santa Clara, CA, USA). Data analysis was performed in the same way as for the colorimetric method, but the dilution factor used in Equation (1) was 1.275 instead of 5.

### 2.5. Chromatographic

The chromatographic method was developed by scientists at Agropur US (Protein Research Center, Le Sueur, MN, USA). A central composite DOE was used to build a response surface model system that would predict NANA hydrolysis reaction outcomes for experimental parameters: acetic acid concentration (1.18 M–2.82 M), reaction temperature (70–90 °C), reaction time (22–218 min), and GMP sample concentration. In each experiment of the design, 200 µL of acid and 200 µL of GMP sample solution were combined and analyzed for the liberation of NANA from GMP. The measured NANA responses were used to build the response surface model optimizing for maximum measured NANA. The design and data analysis were performed using Minitab^®^ (Version 17, Minitab LLC, State College, PA, USA). Parameters were adjusted further using 1.1 M acetic acid at 67.5 °C for a range of 30–360 min with differing concentrations of GMP (1000 µg/mL, 800 µg/mL, 600 µg/mL, and 500 µg/mL). These concentration values were plotted to find where NANA liberation stabilized. An extended time reaction was also assessed to measure the loss of NANA over 20 h.

A modified version of the Agropur US chromatographic method was performed at the Food and Dairy Innovation Center, Boise State University, as follows. The standard addition calibration matrix (0.1200 ± 0.0050 g GMP) and sample (0.0600 ± 0.0010 g GMP) were dissolved in nanopure water on stir plates and brought to a volume of 100.0 mL in volumetric flasks. An NANA (97%, Thermo Scientific, Waltham, MA, USA) standard solution (1 mg/mL) was prepared in nanopure water and stored in the freezer when not in use. From this stock solution (spiking stock #4), a serial dilution (1:4) was used to generate spiking stocks #3, #2, and #1. Five different calibration standards were prepared in microcentrifuge tubes by combining the calibration matrix (500.0 µL) with either one of the spiking stocks or nanopure water (500.0 µL) for the low calibration point. Each calibration standard and duplicate GMP test samples (200.0 µL) were added to 2 mL polypropylene microtubes (Thermo Scientific, Waltham, MA, USA) and aspirated via pipet tip with 2.2 M acetic acid (200.0 µL). The tubes were incubated overnight at 60 °C to hydrolyze NANA from GMP. The following day, samples and standards were evaporated to dryness using a SpeedVac with the temperature set to 65 °C. The samples and standards were reconstituted in a surrogate internal standard (200.0 µL) of ^13^C_3_-NANA (Sigma-Aldrich, Saint Louis, MO, USA) in 50/50 acetonitrile (>99% purity, Fisher Scientific, Pittsburgh, PA, USA)/nanopure water (100 µg/mL), which was stored in the freezer when not in use. The solutions were vortexed until the solid dissolved, and were then sonicated for 10 min. A 50/50 acetonitrile (>99% purity, Fisher Scientific, Pittsburgh, PA, USA)/nanopure water solution (800.0 µL) was added to each tube and vortexed for 10 s. The solutions were syringe-filtered with 0.2 µm nylon filters into HPLC vials.

NANA separation was performed using an Agilent 1260 Infinity II HPLC system (Agilent, Santa Clara, CA, USA) with a quaternary pump, thermostatted column compartment, and split loop autosampler using a Waters Corp XBridge BEH Amide Column, 3.5 µm, 2.1 mm × 100 mm (Waters, Milford, MA, USA). The HPLC injection volume was 10 µL and the flow rate was 1 mL/min with the column temperature set at 30.0 °C. Mobile phase A was 200 mM ammonium formate (Sigma-Aldrich, Saint Louis, MO, USA) acidified to pH 3.3 using formic acid (Sigma-Aldrich, Saint Louis, MO, USA) (A); mobile phase B was acetonitrile (>99% purity, Fisher Scientific, Pittsburgh, PA, USA) (B); and mobile phase C was nanopure water (C). Mobile phase A was held at 5.0% for the entirety of the method. From 0.00 to 1.50 min: (B) = 90.0%, (C) = 5.0%. From 1.50 to 5.10 min: (B) 90.0% to 67.1%, (C) 5.0% to 27.9%. From 5.10 to 5.70 min: (B) 67.1 to 50.0%, (C) 27.9% to 45.0%. From 5.70 to 6.70 min: (B) = 50.0%, (C) = 45.0%. From 6.70 to 6.80 min: (B) 50.0% to 90.0%, (C) 45.0% to 5.0%. From 6.80 to 18.50 min: (B) = 90.0%, (C) = 5.0%. Analyte detection was accomplished with an Agilent G6125C single quadrupole liquid chromatography–mass spectrometry detector system in positive ion mode set to 310.1 *m*/*z* and 313.1 *m*/*z*, capillary voltage positive setpoint: 4000, negative setpoint: 4000, nebulizing gas flow of 7.0 L/min, nebulizer set to 15 psi, gas temperature set to 300 °C, and electrospray ionization as the ion source. MS peaks eluted between 4 and 7 min for both the 310.1 *m*/*z* and 313.1 *m*/*z*. The areas under each curve were integrated, and their ratios were plotted vs. standard concentrations to generate a linear standard addition calibration curve for NANA. The curve was corrected by subtracting the y-intercept from the equation of the line. The equation of the line from the calibration curve was used to calculate the concentration of NANA in each sample (µg/mL). The density of water (1 mg/mL), the dilution factor, and the mass of the GMP sample were used to compute the percentage of NANA per total sample mass (%*w*/*w*).

### 2.6. Method Validation

Five different criteria were used to evaluate each method: time, protein-specificity, linearity, precision, and accuracy. For the time evaluation, the time to complete the entire assay, from sample preparation to data analysis, was determined. This was broken down into active and inactive time for the analyst. Protein-specificity was explored based on the level of specificity the hydrolysis agent (acid or enzyme) had for removing NANA from GMP. Linearity of each assay was derived from the calibration curves generated from NANA standards. The slope, intercept, and coefficient of determination for each regression equation were calculated. The limit of detection (LOD) and limit of quantification (LOQ) were generated from the standard error of the intercept and the slope of each regression equation. The precision of each method was assessed by calculating the average NANA content (%*w*/*w*) on GMP, as well as the standard deviation (SD) and the relative standard deviation (RSD) based on the number of GMP replicates tested. Finally, accuracy was measured by comparing to a reference material (bovine fetuin) for the enzymatic assay or by spike and recovery for the chromatographic approach. The fetuin sample was treated in the same way as the GMP test solution preparation, and the resulting NANA content (%*w*/*w*) was compared to the predicted NANA content. Spike and recovery experiments were performed in triplicate for the chromatographic method. A volume of 50 µL of 1 mg/mL NANA standard was spiked into 1.0 mL filtered GMP test solution. The spiked samples went through the same procedures as the normal GMP test solution preparation.

### 2.7. Statistical Analysis

All statistical analysis was performed using Microsoft 365 Excel (Microsoft, Redmond, WA, USA).

## 3. Results

The measurement parameters used to assess each NANA quantitation method were time, protein-specificity, linearity, precision, and accuracy. These evaluation strategies have been used to review similar methods in the literature [39,40].

### 3.1. Time

**Figure 2:**
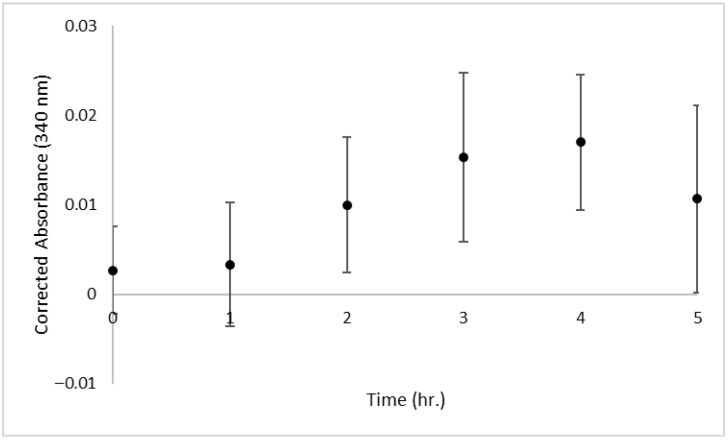
The time-course experiment for enzymatic hydrolysis of *N*-acetylneuraminic acid (NANA) from glycomacropeptide (GMP). UV absorbance at 340 nm over time showed an optimum incubation time of 4 h for maximum hydrolysis of NANA. Standard deviation error bars for n = 3 samples.

The first point of interest is the length of time required to perform each method, including sample preparation, experimentation, waiting periods, and data analysis. The protocol to use the colorimetric/fluorometric kit has two steps with 1 h waiting periods, complemented by multiple timed centrifugation steps and cooling periods. When paired with sample and standard preparation and data analysis, the kit requires a minimum of 4.5 h to complete. The enzymatic kit suggests a hydrolysis time-course experiment prior to running the complete assay. During the time-course experiment, the optimum time for hydrolysis was determined to be 4 h (Figure 2). With the 4 h hydrolysis, the required incubation period of 1 h, the sample and standard preparation required, and analysis of the resulting data, the entire experiment takes approximately 8 h to complete. The chromatographic approach has an overnight hydrolysis period and a drying period that takes at least 2 h to achieve. With the sample preparation and LC-MS quantitation, the chromatographic method requires a total of 31 h, inclusive of 2 h sample preparation, an overnight incubation period, 2 h drying periods, 4 h for LC-MS sample preparation, and 1 h for manual peak integration and data analysis. A summary of protocol times is provided in Table 1.

The fastest method to perform is the colorimetric/fluorometric kit, that takes 4.5 h, followed by the enzymatic protocol at 8 h, and lastly, the chromatographic approach is the most time consuming at 31 h. The majority of the time for each method comes from the hydrolysis reaction, drying, and/or separation times. Each experiment has comparable times where the analyst is in the laboratory, actively conducting the study or analyzing data. The colorimetric/fluorometric method involves 2.5 h of laboratory work and data analysis, the enzymatic method requires 3 h, and the chromatographic approach involves 4 h of scientist effort. These times are based on the current study, where an analyst performed each method three or more times.

### 3.2. Protein-Specificity

NANA hydrolysis requires the appropriate acid strength or enzymatic reaction, proper temperature control, and sufficient time. The characteristics of these parameters are protein dependent, which can make methods tedious and expensive when they are very specific, accurate, and reliable [41]. The colorimetric/fluorometric kit is marketed for quantification of free and total NANA in matrices including serum, saliva, and milk [35]. Similarly, the enzymatic assay is described as determining total NANA content free in solution or bound to glycoproteins or polysaccharides regardless of bond type [36,37]. The enzymatic time-course experiment allowed for maximum release of NANA from GMP to be explored before proceeding with the entirety of the method. The chromatographic approach utilized central composite DOE to model reaction conditions for liberation of NANA from GMP based on empirical measurements. This technique allowed for the maximum amount of NANA detection before degradation by testing hydrolysis time, acid concentration, oven temperature, and GMP sample concentration. The results showed that the maximum amount of NANA was released from GMP when the temperature was held at 67.5 °C for 6 h for a sample consisting of 600 µg/mL GMP and the hydrolysis acid, acetic acid, at a concentration of 1.1 M.

The colorimetric/fluorometric and the enzymatic assays are not sample specific, making their use generic to detect free or bound NANA. The kits can be used for a wide range of applications, but their lack of specificity for NANA detection on a glycosylated protein, like GMP, equates to non-optimal quantitation. Widely variable results and poor quantitation likely originate from the rapid, intense protocol that inconsistently hydrolyzes NANA from GMP. In contrast, the chromatographic approach was specifically designed to cleave and measure NANA on GMP. The central composite DOE was used to determine the exact specifications to achieve maximum NANA hydrolysis and measurement from GMP. The DOE result called for a longer incubation period at a lower temperature with a less concentrated weak acid than the other methods. The chromatographic method was further extended to overnight hydrolysis because it provided a greater level of convenience for the analyst than a 6 h hydrolysis period, but the result was within 0.5% NANA content.

### 3.3. Linearity

Linearity ensures a method is fit for its intended purpose by relating analyte concentration to instrument response. Linearity of each calibration curve was measured via regression analysis. The number of NANA standards used to generate the curve, the concentration, slopes, intercepts, coefficients of determination, LOD, and LOQ, and regression equations for each method are provided in the supplemental materials (Tables S1–S5). A summary of calibration results is listed in Table 2. LOD and LOQ were determined using Equations (2) and (3), respectively.

(2) $$LOD=3.3\times \frac{\sigma }{s}$$

where σ is the standard error of the intercept, and s is the slope.

(3) $$LOQ=10\times \frac{\sigma }{s}$$

where σ is the standard error of the intercept, and s is the slope.

The LOD represents the lowest concentration of NANA the instrument could detect with a certainty of ≥95%. The lowest average LOD of the four methods was 2.13 ± 1.20 µg/mL for the fluorometric, followed by the chromatographic at 10.7 ± 3.3 µg/mL, and the colorimetric at 16.5 ± 11.5 µg/mL, and finally the enzymatic at 52.1 ± 3.2 µg/mL. The LOQ is the lowest value at which a quantified measurement of NANA can be considered accurate based on Equation (3). The lowest quantifiable value was for the fluorometric method at 6.44 ± 3.63 µg/mL, the next lowest was the chromatographic at 32.5 ± 10.0 µg/mL, then the colorimetric at 50.1 ± 34.7 µg/mL, and the highest was the enzymatic at 158 ± 10 µg/mL. The values reported for the enzymatic method are problematic considering none of the five reported sample concentrations are greater than the LOQ. Out of the 12 samples analyzed using the colorimetric method, only 42% of them measured NANA above the LOQ. For the chromatographic method, 80% of samples were above the LOQ. Only the fluorometric method had 100% of samples calculated above the LOQ.

### 3.4. Precision

Precision was assessed to determine the inter-day reproducibility of each measurement. The amount of NANA on GMP was measured as the mass of NANA over the mass of GMP (%*w*/*w*). To report precision, the average, standard deviation (SD), and relative standard deviation (RSD) were calculated for all samples and for those above the LOQ (Table 3).

There was variability in the number of samples included in this study due to the quantity of reagents available in each kit and assay. The colorimetric/fluorometric kit was prepared with sufficient reagents to measure 100 samples and was determined to contain leftover reagents after 100 assays had been run. Three different kits were purchased for the colorimetric/fluorometric assays, but only one kit was viable for use. Therefore, the data included is solely from the second kit. The 12 colorimetric samples were measured across three different days, and the 11 fluorometric samples were also quantified on three different days. Two enzymatic kits were purchased, and each was intended to be used for 25 samples. Neither kit had sufficient reagents to conduct 25 assays, and, of the experiments completed, not all the calibration curves were usable for quantifying NANA content. The usable data was collected on two different days. The chromatographic approach did not have the reagent constraints of the other methods; thus, it was run 10 times for statistical significance. Each experiment was performed on a different day to demonstrate inter-day reproducibility.

The average, standard deviation (SD), and relative standard deviation (RSD) were calculated for each method. The enzymatic method provided the least consistent results with the largest RSD at 23.09%. The fluorometric method was next with an RSD of 18.90%. The colorimetric and chromatographic assays had much lower RSD values of 4.48% and 1.94%, respectively. The chromatographic method had the lowest relative standard deviation among the methods evaluated, providing the most precise measurements.

### 3.5. Accuracy

Each method was evaluated for accuracy to determine which analysis aligned most closely with the actual value of NANA on GMP. Accuracy is typically determined by comparing to a reference method or by using a standard reference material. Neither of these currently exist for the measurement of NANA content. Bovine fetuin (0.5 mg) was provided as a standard reference material for the enzymatic assay and should have yielded approximately 48 nmoles of NANA [38]. When following the procedures for digestion and calculation of nmoles for fetuin, the result was 61.24 nmoles. This value was determined using a calibration curve with an R^2^ value of 0.8168, which was ruled unfit for analysis.

When there is not a standard method or reference material for comparison, accuracy can be assessed by performing a spike and recovery analysis. This approach was used for the chromatographic method. Three different samples were spiked with a known amount of NANA standard, and the percent recovery was measured. The acceptable recovery for this type of validation is within the range from 80 to 120%. Each of the three samples were within the accepted range, and the average recovery was 90.25% (Table 4).

Spike and recovery experiments were successfully performed for the chromatographic approach and were attempted for the colorimetric method. A valid calibration curve could not be produced for the colorimetric method, so the spike and recovery results were discarded. The spike and recovery results for the chromatographic method suggest the accuracy of this approach.

After performing the previously described methods, three distinct hybrid approaches were attempted by combining the acid hydrolysis portion of the chromatographic method with colorimetric detection. The most promising results were from one of the colorimetric kits, but when it was attempted again with another colorimetric kit, it was unsuccessful. Variations on Skoza’s [42] method were trialed, but a spike and recovery analysis performed to validate the results provided values outside of the acceptable range of 80–120%. The hybrid approach was abandoned in favor of the more robust and reliable chromatographic method.

## 4. Discussion

The NANA measurement methods that are available generally employ a combination of hydrolysis and detection of NANA through colorimetric methods. As far back as 1959, Warren measured NANA free in solution and in tissues and fluids obtained from bovine, human, mouse, rat, guinea pig, and chicken sources [34]. This and other similar methods are not well-designed for quantitation of NANA due to limited specificity, poor sensitivity, and their propensity to produce toxic waste [29,34,43]. The commercially available kit used for this work is an improvement from these earlier methods, but it still has drawbacks. This method is rapid to perform, only requiring a total of 4.5 h, where the analyst is actively working for 2.5 h. Another benefit of this method is its precision. For all the samples that were above the LOQ, there was a 4.48% RSD. This was one of the lowest RSD values out of the four methods tested, second only to the chromatographic approach. This kit is not protein-specific, which means that its hydrolysis conditions do not work equally for every protein. While the hydrolysis time is short, the conditions are harsh, which led to variability in the reported NANA content. The LOD for this method is 16.5 ± 11.5 µg/mL, and the LOQ is 50.1 ± 34.7 µg/mL. Over 50% of the samples tested using this method were not within the LOQ. This alone renders this method highly untrustworthy. Based on the issues with linearity and protein-specificity it was deemed unnecessary to proceed with detailed studies on accuracy for this assay. If the goal is to measure NANA on GMP, the benefits of this method do not outweigh the drawbacks.

### 4.1. Fluorometric

The only difference between the fluorometric and colorimetric procedures was that all the standards were diluted tenfold. Therefore, the time required to complete the experiment was still 4.5 h with 2.5 h of active work for the analyst and the lack of protein-specificity was the same. There were a few differences in the results of these two assays. The linearity of the fluorometric assay was its most notable positive feature. It had an LOD of 2.13 ± 1.20 µg/mL and an LOQ of 6.44 ± 3.63 µg/mL. This assay had the lowest LOD and LOQ and is the only method tested where all the sample concentrations of NANA were above the LOQ. This did not translate to the results on precision. The RSD was nearly 20%, which showed that the results were not clustered near the mean of 7.26% NANA. This variation indicates that this method does not provide consistent results for the same reference material, and thus cannot be considered reliable. Spike and recovery studies to test accuracy were not attempted due to this level of unreliability.

### 4.2. Enzymatic

A variety of enzymatic assays were explored in the 1960s and 1970s [26,28]. These often involved the conversion of NANA into a series of products including hydrogen peroxide or pyruvate that were able to be detected and measured using UV-Vis spectroscopy or biosensors [26]. The method used here utilized a neuraminidase enzyme, which can cleave α(2→3,6,8,9) bonds. This should allow for the cleavage of any bonded NANA. The time-course experiment was performed to determine the best hydrolysis parameters for GMP, which demonstrated a level of protein-specificity. Having a 4 h hydrolysis period along with sample preparation and other procedures resulted in this method requiring about eight hr., where for three hr. the analyst is actively working. This can be completed in a standard day of work, but does not have the speed of the colorimetric and fluorometric assays. Drawbacks of this method began with the linearity results, which were by far the highest of the four methods at an LOD (52.1 ± 3.2 µg/mL) and LOQ (158 ± 10 µg/mL). None of the values reported for the five samples tested on this method were within the LOQ, which causes this method to be unusable for this application. In addition, this method had the worst precision with an RSD of nearly 25%. The accuracy was tested using the provided bovine fetuin standard, and the reported NANA value was over 125% of the expected value. This method may have had increased protein-specificity due to the initial time-course experiment, but it does not have sufficient linearity, precision, or accuracy to be recommended for use.

### 4.3. Chromatographic

Chromatographic assays for measuring NANA content became prevalent in the literature reports in the early 1980s [26]. One of the key features of the chromatographic method used for this work was the DOE for the hydrolysis parameters. The protein-specificity of the chromatographic method is unmatched compared to the other three methods tested. Hydrolysis temperatures and times were evaluated here, and previous work performed by Joseph Hale at Agropur’s Protein Research Center determined the type of acid, concentration of acid, and concentration of sample. One of the highlights of this method is its precision, which was the most superior of any of the methods. The RSD was 1.94% for the samples that were above the LOQ, meaning the sample values were tightly grouped around the mean. Measured NANA content was reproducible. This method was also tested for accuracy via spike and recovery experiments. Each of the triplicate runs was within the accepted tolerance of 80–120%, and the average recovery was 90.25%. Having an accurate and reliable method is necessary for standardization across the industry. The chromatographic approach had the second best LOD (10.7 ± 3.3 µg/mL) and LOQ (32.5 ± 10.0 µg/mL) after the fluorometric method. Out of the 10 samples tested, 8 of them were within the LOQ. This is a limitataion of the assay, though it still performed sufficiently 80% of the time. The biggest drawback is the length of time required to perform this experiment. With the overnight method, a total of 31 h are needed with 4 of those being active time for the analyst. Overall, the benefits of this assay far outweigh the drawbacks, making it the best option for a standard method.

## 5. Conclusions

The results of this work compared facets of four different methods used to hydrolyze and quantify NANA on GMP. When considering the length of time, protein-specificity, linearity, precision, and accuracy of each method, the chromatographic approach was unequivocally the best for this purpose. Scientists at Agropur US developed the chromatography method as a robust and accurate means to quantify NANA on GMP. The protocol was altered slightly to require a total of 31 h. to complete, during which the analyst is actively involved for 4 h. Out of the 10 GMP samples examined, 8 of the measured values were above the 32.5 ± 10 μg/mL LOQ threshold for NANA. The chromatography method is the only one specifically tailored by DOE to the GMP matrix, and it also provided the lowest RSD across all methods at 1.94%. The chromatography method did not have the issue of inconsistency between kits, and when testing for accuracy by spike recovery experiments, the average NANA recovery was 90.25%. Although the chromatographic method requires more time to perform than other methods, and the LOQ was determined to be satisfied for 80% of test cases, the specificity of this method for NANA on GMP demonstrated superior precision and accuracy, setting it apart from the other methods. The ability to measure NANA on GMP is critical for consistent formulation of existing and new products within the dairy industry. At this time, the functional and bioactive properties of NANA on GMP are largely underutilized in GMP’s limited application as a protein source for PKU-safe foods. With implementation of this chromatographic method, the functionality and bioactivity could be harnessed for the development of novel products.

## Figures and Tables

**Table 1:** Approximate time required to complete each method for quantitation of *N*-acetylneuraminic acid (NANA) in glycomacropeptide (GMP). Active time is the duration an analyst spends performing a test method step excluding incubations and waiting.

Method	* Colorimetric	* Fluorometric	* Enzymatic	* Chromatographic
Sample Preparation	**2 h**	**2 h**	**2.5 h**	**3 h**
Reaction or Drying	2 h	2 h	5 h	24 h
Detection	30 s	30 s	30 s	3 h
Data Analysis	**0.5 h**	**0.5 h**	**0.5 h**	**1 h**
Total	4.5 h	4.5 h	8 h	31 h
Total Active Time	**2.5 h**	**2.5 h**	**3 h**	**4 h**

* Active time for the analyst is in bold.

**Table 2:** Summary of method linearity including number of standards, range of concentration, number of calibration curves, and the average ± standard deviation for slope, intercept, coefficient of determination, LOD, and LOQ.

Method	Colorimetric	Fluorometric	Enzymatic	Chromatographic
Number of standards (n)	4	4	4	5
Range of concentration (µg/mL)	0–309	0–30.9	0–309	0–485.0
Number of calibration curves	3	3	2	10
The slope of the regression equation	0.0017 ± 0.0001	39.747 ± 6.741	0.0003 ± 0.0000	9.6162 ± 0.4943
The intercept of the regression equation	−0.0056 ± 0.0106	71.532 ± 44.610	−0.0029 ± 0.0113	0.387 ± 0.030
Coefficient of determination	0.9979 ± 0.0024	0.9969 ± 0.0032	0.9858 ± 0.0113	0.9992 ± 0.0006
Limit of detection (µg/mL)	16.5 ± 11.5	2.13 ± 1.20	52.1 ± 3.2	10.7 ± 3.3
Limit of quantification (µg/mL)	50.1 ± 34.7	6.44 ± 3.63	158 ± 10	32.5 ± 10.0

**Table 3:** Inter-day reproducibility of NANA measurement of the mass of NANA over the mass of GMP in %*w*/*w* based on number of measurements, average, standard deviation (SD), and relative standard deviation (RSD).

Method	* Colorimetric	* Fluorometric	* Enzymatic	* Chromatographic
Number of observations (n)	12	11	5	10
Average	**5.25** (5.11)	**7.26**	(14.09)	**6.18** (6.17)
SD	**0.24** (0.27)	**1.37**	(3.25)	**0.12** (0.15)
RSD (%)	**4.48** (5.19)	**18.90**	(23.09)	**1.94** (2.44)

* Bold values represent numbers within the LOQ, and numbers in parentheses represent the average of all samples.

**Table 4:** *N*-acetylneuraminic acid (NANA) spike and recovery for chromatographic method.

Sample	Measured NANA (%*w*/*w*)	Recovery (%)
1	6.25	96.32
2	5.94	81.18
3	6.44	93.24
Average	6.21	90.25

## Data Availability

The original contributions presented in this study are included in the article/Supplementary Material. Further inquiries can be directed to the corresponding author.

## Supplementary Materials

The following supporting information can be downloaded at: https://www.mdpi.com/article/10.3390/foods14223939/s1, Table S1: Linearity of colorimetric method; Table S2: Linearity of fluorometric method; Table S3: Linearity of enzymatic method; Table S4: Linearity of chromatographic method; Table S5: Regression equations for each method.

## Abbreviations

The following abbreviations are used in this manuscript:

*beta*-NADH	*beta*-nicotinamide adenine dinucleotide
DI	Deionized
DMSO	Dimethyl sulfoxide
DOE	Design of Experiments
Gal	Galactose
GalNAc	*N*-acetylgalactosamine
GC	Gas chromatography
GC-MS	Gas chromatography-mass spectrometry
GMP	Glycomacropeptide
HPLC	High-performance liquid chromatography
HPLC-MS	High-performance liquid chromatography–mass spectrometry
LOD	Limit of detection
LOQ	Limit of quantification
NANA	*N*-acetylneuraminic acid
OD	Optical density
Phe	Phenylalanine
PKU	Phenylketonuria
RSD	Relative standard deviation
SD	Standard deviation
Ser	Serine
TCA	Trichloroacetic acid
Thr	Threonine
Tris-HCl	Tris(hydroxymethyl)aminomethane hydrochloride
