# Supplementary material for: Evaluation of Methods to Quantify Sialic Acid on Glycomacropeptide
Source: Foods. 2025 Nov 18;14(22):3939. doi: 10.3390/foods14223939 (PMC12651296; doi:10.3390/foods14223939)
Supplement: Supplementary file 1 [file foods-14-03939-s001.zip › foods-3966436-supplementary.pdf]

Supplementary Materials

Table S1. Linearity of Colorimetric Method

Range of concentration (μg/mL) [n=4]	0-309		
The slope of the regression equation	0.0018	0.0016	0.0016
The intercept of the regression equation	-0.0182	-0.0062	0.0077
Correlation coefficient	0.9945	0.9993	0.9998
Limit of detection (μg/mL)	32.3	11.8	5.46
Limit of quantification (μg/mL)	97.9	35.7	16.5

Table S2. Linearity of Fluorometric Method

Range of concentration (μg/mL) [n=4]	0-30.9		
The slope of the regression equation	45.725	43.189	30.326
The intercept of the regression equation	63.507	129.91	21.628
Correlation coefficient	0.999	0.9923	0.9993
Limit of detection (μg/mL)	1.39	3.81	1.17
Limit of quantification (μg/mL)	4.22	11.6	3.54

Table S3. Linearity of Enzymatic Method

Range of concentration (μg/mL) [n=4]	0-309	
The slope of the regression equation	0.0003	0.0003
The intercept of the regression equation	0.0084	-0.0141
Correlation coefficient	0.984	0.9875
Limit of detection (μg/mL)	55.3	48.9
Limit of quantification (μg/mL)	168	148

Table S4. Linearity of Chromatographic Method

Range of concentration (μg/mL) [n=4]	0-485.0									
The slope of the regression equation	9.650	9.821	9.907	9.465	9.623	9.403	9.501	8.568	10.68	9.545
The intercept of the regression equation	0.3678	0.4326	0.3286	0.3880	0.3994	0.4177	0.3950	0.3650	0.3587	0.4125
Correlation coefficient	0.9996	0.9995	0.9976	0.9996	0.9994	0.9991	0.9992	0.999	0.9996	0.9993
Limit of detection (μg/mL)	7.854	8.549	19.14	7.377	9.367	11.31	10.99	13.29	8.318	10.98
Limit of quantification (μg/mL)	23.80	25.91	58.00	22.35	28.38	34.28	33.29	40.28	25.20	33.27

Table S5. Regression Equations for Each Method

Colorimetric	Fluorometric	Enzymatic	Chromatographic
y = 0.0018x - 0.0182	y = 45.725x + 63.057	y = 0.0003x + 0.0084	y = 9.6504x + 0.3678
y = 0.0016x - 0.0062	y = 43.189x + 129.91	y = 0.0003x - 0.0141	y = 9.8208x + 0.4326
y = 0.0016x + 0.0077	y = 30.326x + 21.628		y = 9.9073x + 0.3286
			y = 9.4646x + 0.388
			y = 9.6234x + 0.3994
			y = 9.4033x + 0.4177
			y = 9.5011x + 0.395
			y = 8.5677x + 0.365
			y = 10.679x + 0.3587
			y = 9.5447x + 0.4125
